# Fluorinated Polymers as Smart Materials for Advanced Biomedical Applications

**DOI:** 10.3390/polym10020161

**Published:** 2018-02-08

**Authors:** Vanessa F. Cardoso, Daniela M. Correia, Clarisse Ribeiro, Margarida M. Fernandes, Senentxu Lanceros-Méndez

**Affiliations:** 1 Centro/Departamento de Física, Universidade do Minho, 4710-057 Braga, Portugal; cribeiro@fisica.uminho.pt (C.R.); margaridafernandes@fisica.uminho.pt (M.M.F.); 2 CMEMS-UMinho, Universidade do Minho, DEI, 4800-058 Guimaraes, Portugal; 3 Departamento de Química e CQ-VR, Universidade de Trás-os-Montes e Alto Douro, 5001-801 Vila Real, Portugal; dcorreia@utad.pt; 4 BCMaterials, Basque Center for Materials, Applications and Nanostructures, UPV/EHU Science Park, 48940 Leioa, Spain; senentxu.lanceros@bcmaterials.net; 5 CEB—Centre of Biological Engineering, University of Minho, 4710-057 Braga, Portugal; 6 IKERBASQUE, Basque Foundation for Science, 48013 Bilbao, Spain

**Keywords:** fluorinated polymers, electroactive, piezoelectric, poly(vinylidene fluoride), biomedical applications

## Abstract

Fluorinated polymers constitute a unique class of materials that exhibit a combination of suitable properties for a wide range of applications, which mainly arise from their outstanding chemical resistance, thermal stability, low friction coefficients and electrical properties. Furthermore, those presenting stimuli-responsive properties have found widespread industrial and commercial applications, based on their ability to change in a controlled fashion one or more of their physicochemical properties, in response to single or multiple external stimuli such as light, temperature, electrical and magnetic fields, pH and/or biological signals. In particular, some fluorinated polymers have been intensively investigated and applied due to their piezoelectric, pyroelectric and ferroelectric properties in biomedical applications including controlled drug delivery systems, tissue engineering, microfluidic and artificial muscle actuators, among others. This review summarizes the main characteristics, microstructures and biomedical applications of electroactive fluorinated polymers.

## 1. Introduction

Since the discovery of polytetrafluoroethylene (PTFE) in 1938, fluorinated polymers have been the subject of intense research and development due to the increasing demand on improved performing materials [1,2,3]. Fluorinated polymers constitute a unique class of materials with a combination of interesting properties owing to their chemical backbone, which is formed by strong carbon-carbon bonds (~340 kJ·min^−1^) and stable carbon-fluorine bonds (~485 kJ·min^−1^), and also because of the special electronic structure of the fluorine element characterized by high electronegativity, low polarizability and small van del Waals radius (1.32 Å) [4]. Due to those intrinsic characteristics, polymers containing fluorinated groups show high thermal stability, weather stability, low coefficient of friction and lower surface energy when compared to their non-fluorinated counterparts [5,6,7]. Moreover, fluorinated polymers are chemically stable and inert or relatively unreactive. Since fluorine induces more stability compared to chloride, the reactivity usually decreases with increasing fluorine content. Fluoropolymers can start to produce toxic air contaminants at, or slightly above, their recommended processing temperatures. Thus, exhaust ventilation is recommended during processing operations at high temperatures [8]. Fluorinated polymers usually present inferior mechanical properties [9]. To overcome this limitation, various fluorinated polymers with unique and exceptional variable set of properties were developed during the last decades.

Typical commercially available fluorinated polymers present high molecular weights, providing superior performance in many fields, including buildings, automotive, aircraft, aerospace, aeronautics, chemistry, textile, energy, microelectronics and biomedical applications, among others [9,10,11,12,13,14]. They are commonly divided in two types: perfluoropolymers where all hydrogen atoms in the analogous hydrocarbon polymer structures are replaced by fluorine atoms, and partially fluorinated polymers where both hydrogen and fluorine atoms remain in the polymer structures along with additional elements in specific cases [2]. Most of them are produced from monomers of tetrafluoroethylene (TFE), chlorotrifluoroethylene (CTFE), vinylidene fluoride (VDF) and vinyl fluoride (VF) (Table 1) [15]. TFE is commonly used as monomer to produce a variety of perfluoropolymers, such as PTFE. Besides, TFE is also used for the production of other monomers, such a hexafluoropropylene (HFP) and perfluoro(propyl vinyl ether) (PPVE).

PTFE, commercially known as Teflon, shows one of the highest thermal stability among organic materials, with a continuous-use temperature below 260 °C and it is generally synthesized with a very high molecular weight to obtain adequate mechanical properties [41,42]. Thus, PTFE is hardly processed by traditional methods used in typical polymers such as injection moulding and extrusion moulding because of its extremely high melt viscosity. Moreover, it does not dissolve or swell in most chemicals and so, machining techniques are commonly used to process PTFE. Therefore, PTFE has an exceptional chemical resistance, except for some extreme conditions related to molten alkali metals or elemental fluorine [2]. It presents a low dielectric constant due to the highly symmetrical structure of the macromolecules. Additionally, PTFE products have the particularity of repelling water, oil and adhesives, which is interesting for some applications [43,44,45]. Other appealing perfluoropolymers are tetrafluoroethylene-hexafluoropropylene (FEP) and perfluoroalkoxy (PFA), which are melt processable copolymers with properties similar to PTFE [46,47]. FEP and PFA are obtained from the copolymerization of TFE with HFP and PPVE, respectively. Both polymers show lower molecular weights than PTFE, in particular FEP, and can be easily processed by conventional polymer processing methods due to the resulting lower melt viscosities [48]. In terms of chemical and weather resistances and electrical properties, FEP and PFA are quite similar to PTFE (Table 1) [49]. Moreover, while PFA thin films present higher optical transparency than PTFE, FEP surfaces are more easily modified to increase adhesive bonding [50,51,52]. Amorphous perfluoropolymers such as Cytop, Teflon AF and Hyflon AD were introduced later and present unparalleled optical transparency (ranging from the ultraviolet to the infrared region), low refractive index, and the lowest dielectric constant of all known polymers, leading to excellent reflective properties [53,54]. They feature similar chemical, thermal and electrical properties to crystalline perfluoropolymers but with better mechanical properties and they are also soluble in fluorinated solvents [55,56]. However, the high prices of amorphous perfluoropolymers restricts their use to some specific applications, mostly in optical lenses and fibres [57,58,59].

Nevertheless, because of the still poor mechanical properties of perfluoropolymers, partially fluorinated polymers, mostly obtained from fluoroethylene, with higher polarity and consequently improved mechanical properties appeared as good alternative. Although they have inferior chemical resistance compared to perfluoropolymers, partially fluorinated polymers are soluble in common solvents. Examples of partially fluorinated polymers are poly(chlorotrifluoroethylene) (PCTFE), ethylene-tetrafluoroethylene copolymer (ETFE), poly(vinyl fluoride) (PVF), and poly(vinylidene fluoride) (PVDF). The simple replacement of one fluorine atom of PTFE with chlorine results in lower crystallinity, lower melting temperature and better mechanical properties of PCTFE, which, however, has an unfavourable impact on its thermal stability, chemical resistance and electrical properties [60,61]. Nevertheless, PCTFE is easily melted and processed and the products are more transparent than PTFE [2]. Also, PCTFE is the fluoropolymer with the lowest moisture permeability [62]. However, the higher price of PCTFE compared to PTFE limited its use to applications where PTFE cannot meet high performance requirements. ETFE, in turn, contained alternating TFE and ethylene units in the polymer main chain [63]. This partially fluorinated polymer can be processed by all thermopolymer processing techniques and presents enhanced mechanical properties compared to other TFE copolymers [11,64]. Moreover, although it has medium chemical resistance, ETFE shows good wear resistance and high flexibility [65,66]. In the case of PVF, a single hydrogen atom is replaced by a fluorine atom in the polyethylene polymer main chain. As a result, PVF is characterized by a lower crystallinity than PTFE, which results in superior mechanical properties. It also presents excellent weather stability and is highly transparent to radiation between the ultraviolet and near-infrared regions [67,68]. However, the low thermal stability of PVF makes it difficult to process by traditional processing methods, and so, plasticizers and stabilizers are usually added [69,70]. The free radical polymerization of VDF results in the partially fluorinated PVDF polymer. PVDF features similar thermal and weather stabilities than other fluorinated polymers and shows good resistance to most organic and inorganic chemicals [71]. Moreover, PVDF has a significantly lower melting temperature compared to other fluorinated polymers, it presents good mechanical properties, high dielectric response and it can crystallize in various crystalline phases that are correlated to different chain conformations [72]. Nevertheless, it should be noted that PVDF is vulnerable to dehydrofluorination and undergoes nucleophilic attack when treated with strong bases, such as sodium or potassium hydroxide [73,74]. A property that turns PVDF to an extremely interesting polymer for a wide range of advanced biotechnological applications is the strong piezoelectricity presented by its polar β-phase [75,76]. In fact, PVDF, as well as its copolymers, are smart materials with the ability to vary in shape and size when subject to an electric field in a controllable and reproducible way; and reversibly to convert mechanical strain into electrical signals. Therefore, a variety of processing techniques have been developed to produce PVDF-based polymers in the β-phase and with different structures and morphologies, such as films [76,77], membranes [78,79], and fibres [80,81], among others, in order to meet specific applications requirements.

An interesting characteristic related to fluorinated polymers is their superwetting properties. These polymers show the ability to promote microtextured surfaces due to the formation of a stable air-liquid interface, inducing special features on the material [82]. Thus, the creation of superhydrophobic surfaces [83,84], superhydrophilic surfaces [84], superamphiphobic surfaces [85], and directional liquid-transfer surfaces [86], as well as some multifunctional surfaces combining superwettability with adhesion or optical properties [87,88] may be achieved. All these examples exhibit multiscale structures in a biomimetic approach that combine these unique configurations and the intrinsic material properties. These wetting features impart the material advanced properties for a wide range of applications. For example, superhydrophobic surfaces obtained by copper and silicon micro-and nanostructured substrates modified with fluorinated molecules have been developed for anti-icing applications [89]. For anti-fogging or heat transfer purposes, silicon-based materials modified with fluorinated molecules have been developed [90,91]. For sensor development, silicon nanowire arrays modified with fluorinated molecules results in superhydrophobic materials [92]. Similarly, Teflon porous membranes lubricated with perfluorinated fluids give rise to superoleophilic and slippery surfaces [93]. Also used in energy conversion devices, fluorinated polymers have been widely applied as materials in lithium batteries, fuel cells, and solar cells, among others [94].

This review summarizes the main properties of PVDF-based polymers, indicating the different processing strategies to obtain different structures and morphologies as well as some of the most interesting potential applications in the biomedical field.

## 2. Poly(vinylidene fluoride) and Its Copolymers

Poly(vinylidene fluoride) (PVDF) and its copolymers hold unique physicochemical and electrical properties, which makes them the most commonly used fluorinated polymers for an increasing number of advanced applications [95,96,97]. PVDF is a highly non-reactive, semi-crystalline polymer constituted by long chain macromolecules, composed of approximately 59 wt % fluorine and 3 wt % hydrogen, obtained through the polymerization of the monomer vinylidene fluoride. Different conformational structures of PVDF may be obtained depending on polymerization reaction and crystallization conditions. The temperature at which the reaction takes place is recognized as a key factor in the process, influencing the content of head-to-head chains in the polymer and its crystallinity [98,99].

**Figure 1:**
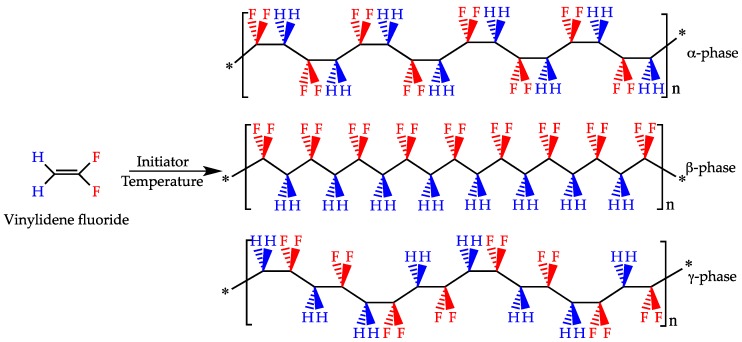
Free radical vinyl polymerization of the monomer vinylidene fluoride to obtain PVDF. Different chain conformations of PVDF in the apolar α-phase and in the polar β- and γ-phases.

At least five polymorphic modifications may be found in the PVDF crystalline phase (identified as α, β, γ, δ and ε), which are defined by the conformational structure of the chains and the macromolecule packing in the unit cells of their crystallites [72,100]. The different chain conformations are designated as all trans (TTT) planar zigzag for the β-phase, TGTG′ (trans-gauche-trans-gauche) for the α- and δ-phases and T3GT3G′ for γ- and ε-phases [72,100]. As shown in Figure 1, where the most investigated PVDF phases are depicted, the position of the fluorine atoms in the molecular chain dictates the phase of the polymer. The high electronegativity of fluorine atoms generates a strong electrical dipole moment of the monomer unit [101,102]. The β- and γ-phases hold the highest dipolar moment per unit cell and thus are known to possess piezoelectricity, while the other three phases (α, δ and ε) are apolar due to antiparallel packing of the dipoles within the unit cell [103]. Nevertheless, the α-phase of PVDF can be transformed into the β-phase and, thus, into the electroactive phase by applying an additional treatment such as stretching, high pressure or polarization. On the other hand, melt crystallization may lead back to the formation of the apolar α-phase [104].

Each phase of PVDF imparts different properties to the polymer but other characteristics such as the molecular weight, molecular weight distribution and extent of irregularities along the polymer chain also play an important role. Among the most widely reported properties of PVDF are the piezoelectricity, the large dielectric constant, and the pyroelectric and ferroelectric effect. Moreover, properties such as improved mechanical strength, wear resistance, and creep resistance when compared with other fluorinated polymers such as PTFE and FEP, have also been reported [2]. These properties are very important to consider the development of smart materials for advanced applications [73,74].

Piezoelectric materials can react to changes in their environment, converting electrical energy to mechanical energy, and vice versa. This means that under mechanical solicitations the charge at the surface of a piezoelectric material varies without the need for an additional energy source or electrodes [105]. This strategy has been applied in tissue engineering applications for the development of scaffolds that support the growth, proliferation and differentiation of different cells such as osteoblast, myoblasts and fibroblasts [106,107,108]. More details on this topic are summarized elsewhere [13]. PVDF also shows pyroelectricity that indicates the capability of PVDF to generate electricity though the application of heat or vice versa. As a ferroelectric material, PVDF also shows a spontaneous electric polarization that can be switched by the application of an external electric field, resulting in a polarization-field hysteresis loop [109].

Due to the electroactive properties, materials comprising the β- and γ-phases of PVDF are the most required for advanced applications. The higher the piezoelectric response, the better for many applications, thus, strategies to improve it have been widely applied. One of the approaches involves the development of PVDF copolymers. Despite the fact that copolymer unit structures are less polar than those of pure PVDF, some copolymers show a higher crystallinity, resulting in higher piezoelectric responses [110]. Other properties, such as glass transition temperature, melting point, stability, permeability, elasticity, and chemical reactivity may also be changed as a result of copolymerization [111]. Poly(vinylidene fluoride-*co*-trifluoroethylene) (P(VDF-TrFE)), poly(vinylidene fluoride-*co*-tetrafluoroethylene) (P(VDF-TFE)), poly(vinylidene fluoride-*co*-hexafluoropropylene) (P(VDF-HFP)) and poly(vinylidene fluoride-*co*-chlorotrifluoroethylene) (P(VDF-CTFE)) are important PVDF copolymers used for advanced functional applications (Table 2).

One of the most studied copolymers is P(VDF-TrFE) that, for specific TrFE contents, crystallizes in the ferroelectric β-phase, which means that P(VDF-TrFE) does not need any further treatment to achieve the electroactive phase, on the contrary to PVDF homopolymer. The addition of the TrFE monomer unit induces a large steric hindrance that favours the all-trans conformation [120] and a higher degree of crystallinity when compared with PVDF. This results in a higher electromechanical coupling factor, *k*, and thus, a higher efficiency in mechanical to electrical conversion [72]. 

Through the incorporation of the amorphous phase of the chemically inert HFP on PVDF, a P(VDF-HFP) copolymer with hydrophobic properties and good mechanical properties may be obtained. An increase in the fluorine content is the reason for the increased hydrophobicity [133]. This copolymer possesses lower crystallinity when compared to PVDF, due to the presence of CF_3_ groups and it has been mainly studied for applications in the area of rechargeable lithium batteries [130,131,132] and for the production of membranes for organophilic pervaporation [128,129]. Nevertheless, the ferroelectric properties of this copolymer are highly dependent on the material processing conditions [72].

Through the introduction of CTFE in the PVDF polymer chain, the copolymer P(VDF-CTFE) is obtained, whose final properties entirely depend on the CTFE content. The semicrystalline state may only be achieved for a CTFE content lower than 16 mol %, while the amorphous state is induced with higher CTFE concentrations [111]. Nevertheless, the insertion of these groups makes the structure loose, facilitating the orientation of dipoles under an external electric field and imparting a piezoelectric constant of 140 pC·N^−1^ (|*d*_33_|), much higher than PVDF homopolymer.

As indicated in Table 2, mainly PVDF and P(VDF-TrFE) have been used as active smart materials, especially in tissue engineering for cell stimulation, while the other two have been mostly used as passive platforms for advanced applications, such as in the case of membranes for distillation. Despite the fact that these copolymers possess even high electromechanical responses when compared to PVDF and P(VDF-TrFE) they require a higher driving electric field, which limits their application in certain application areas of smart materials [111].

The herein presented remarkable properties of PVDF and its copolymers make this class of polymers a focus of interest among the research community for advanced applications in several scientific areas. In fact, these still possess the highest electromechanical responses over a broad temperature range among the known natural and synthetic organic materials.

### Processing Techniques and Resulting Structures

The design of PVDF-based structures plays a key role in determining the suitability of the materials for several applications and tuning their properties for the intended purposes. As a result, different methodologies are available to produce materials into different designs and morphologies in order to meet specific requirements of applications that can vary from drug delivery [134], tissue engineering [135,136], sensors and actuators [77,137], medical device instrumentation [138], microfluidic systems [139], membranes for filtration and environmental engineering [140,141] and energy harvesting [39], among others. Thus, depending on the processing method, PVDF-based materials in the electroactive β-phase can be obtained in the form of films [75,142], porous films [143,144,145], fibres [136] and microspheres [146] (Table 3).

PVDF-based films with a controllable thickness can be obtained by several methods such as doctor blade, spin-coating and printing technologies (inject printing, screen printing and spray printing). Films in the electroactive β-phase with thicknesses ranging from few to hundreds of micrometres can be obtained by the doctor blade process using post-thermal treatment that does not exceeds 70 °C. For higher temperatures or above the melting temperature of the polymer, the crystallization occurs mainly in the apolar α-phase due to the rapid evaporation rate of the solvent [149,157,167]. In this case, mechanical stretching of α-PVDF film at a specific temperature induces the transition to β-phase [72]. Moreover, electrical poling favours the orientation of the dipoles along the intense applied electrical field direction, supporting the crystalline configuration change into the β-phase [168]. In turn, P(VDF-TrFE) always crystallizes in the electroactive β-phase when in specific molar ratios (VDF content between 50% and 80%) [72]. PVDF-based films with thicknesses ranging from hundreds of nanometres to dozens of micrometres and with high structural uniformity can be obtained by spin coating [75,76,77,150,151,152]. The control of the film properties can be performed by varying the polymer concentration, the rotational speed of the spin coater and the temperature of crystallization [75,150]. This technique presents the advantages of allowing the direct deposition of the polymer film on the desired substrate, as occurs with the printing techniques. In fact, films based on PVDF and its copolymers can also be produced by printing technologies, including inkjet, screen and spray printing after properly adjusting the viscosity of the polymer solution [153,156]. 

Porous structures can be obtained in the electroactive β-phase by different methods such as non-solvent-induced phase separation (NIPS) [78,135,157,158,169], temperature-induced phase separation (TIPS) [149,159,160], solvent-casting particulate leaching [144], solvent-casting 3D nylon template [144] and freeze extraction with a 3D poly(vinyl alcohol) (PVA) template [144]. Moreover, patterned porous PVDF-based films can also be obtained by replica moulding using specific templates such as polydimethylsiloxane (PDMS) moulds [145], among others. The NIPS method involves the use of a non-solvent to induce a phase separation either by liquid-liquid or solid-liquid demixing through the immersion of the polymer solution in a coagulation bath containing the non-solvent, originating a polymer-rich phase. This phase determines the microstructure and morphology of the porous film [78,157]. The phase separation of the polymer solution can be also induced by TIPS. In this method, the selection of the proper solvent is a crucial step to control the pore size, since the compatibility of the polymer with the solvent affects the thermodynamic properties of the solution [160]. The major advantages of TIPS are associated with the low tendency for defect formation, high degrees of porosity and effective control of the pore size [160]. Within the methods of processing porous films, freeze extraction using a 3D template induces the formation of interconnected porous with a reproducible morphology, with the pore size being controlled by the size of the sacrificial material [144]. Moreover, it is reported that using a salt (such as sodium chloride (NaCl)) leaching method, PVDF porous structures can present pores with dimensions of hundreds of micrometres [144]. Using nylon templates, the pore size is guided by the space between the filaments and its geometry and porous structures with pores ranging from dozens to hundreds of micrometres can be obtained. Finally, complex patterned porous microstructures can also be obtained by replica moulding using moulds featuring the inverse desired structure [145]. 

Electrospinning is a process that allows obtaining effectively random and aligned fibres using a sufficiently conductive solvent and a static or rotating collector, respectively, with diameters ranging from hundreds of nanometres to dozens of micrometres [136,161,162,163]. In this method, the change of solution properties (such as concentration, viscosity and surface tension) and processing parameters (such as flow rate, needle diameter, distance of needle to collector and applied voltage) allows to achieve and tune the properties of the continuous and charged jet [136]. The crystallization process of PVDF occurs predominantly in the β-phase using high voltage or high stretching ratio of the jets. Furthermore, the processing conditions and solvent used influence other characteristics such as fibre average diameter, specific surface area, surface tension and mechanical properties [170]. The use of stronger applied electrical fields during the process induces the distribution of the dipolar moments along the fibre axis and the electrostatic attractive forces integrate along the length of the fibre segments [171]. The interesting characteristics of the fibres, namely the controllable pore structure, smaller pores and a high surface area are of paramount importance to be successfully applied.

Regarding microspheres, they can be produced by several methods such as phase separation or precipitation, emulsion/solvent evaporation and electrospray [146]. Nevertheless, only few studies report the production of PVDF-based microspheres. It was demonstrated that electrospray is a versatile method to produce PVDF microspheres in the electroactive β-phase with an average diameter between hundreds of nanometres and few micrometres [146,165]. Superhydrophobic PVDF-based microspheres with an average diameter of few micrometres were also obtained by the gelation technique [166]. 

As it was briefly mentioned, various methods promote the smart β-phase formation. It is noteworthy that the amount of β-phase and, therefore, the piezoelectric response can also be enhanced by the application of an additional electrical poling process due to the alignment of the dipoles of the β-phase material inducing also a slight increase of the dielectric constant of the material [172]. Furthermore, the β-phase can also be enhanced with the incorporation of fillers (e.g., cellulose, carbon nanotubes, ferrite, zeolites) into the polymer solution [173]. The filler addition can alter the mechanical, thermal and, thus, the electrical conductivity of the material. Additional functionality, such as magnetoelectric and photocatalytic materials, can also be added to PVDF-based structures by the introduction of functional fillers such as magnetic nanoparticles (Terfenol-D, iron oxide, cobalt ferrites) and titanium dioxide particles, respectively [165,174].

## 3. Representative Biomedical Applications

### 3.1. Medical Device Instrumentation

PVDF and its copolymers are among the class of high-performance polymers with remarkable piezoelectric and ferroelectric polymers. Considering further properties such as flexibility, robustness, easy conformability and lightness, makes these polymers suitable for the development of medical device instrumentation. In its piezoelectric form, PVDF can be used to develop sensors and actuators including artificial muscles [175,176,177], medical imaging [178,179], smart skins [180] and underwater acoustic transducers [181], among others. 

Since long, the fabrication of electromagnetic motors and actuators have enabled an impressive array of robotic and prosthetic devices for muscle and bone therapy. However, these are heavy and do not always fulfil some specifications required for an efficient treatment. The development of PVDF-based actuators has emerged as an alternative for these applications and has been increasingly studied and applied where a muscle-like response is desirable, including in medical devices, prostheses and robotics, among others [175,176]. For example, P(VDF-TrFE) electrospun nanofibres able to be twisted and coiled have been considered a good platform for mimicking muscle structure [178]. 

In the medical and biological imaging field (above 15 MHz frequencies), PVDF and its copolymers have been considered alternative materials to conventional ceramics such as lead zirconate titanate (PZT). PVDF-based polymers possess low acoustical impedance and flexible constitution, addressing the limitations of high acoustical impedance and brittleness of PZT [182]. PVDF hydrophones have long been used for ultrasound imaging and different transducer configurations have been adopted in the development of PVDF-based devices [178,179]. This type of transducers is indeed considered suitable for acquiring ultrasound biomicroscope images of a wide range of human tissues, showing outstanding definition of the tissues [182]. PVDF transducers have been used from its early beginnings for thyroid [178,183], breast [183,184] and skin melanoma imaging [185,186,187], among others. Despite its acoustic power limitation that restricts the transducer’s penetration in depth, it allows excellent definition on “near-surface” areas. Moreover, a micromachined ultrasonic transducer has also been used to measure the mechanical properties of bio cells using piezoelectric PVDF films [188]. By taking advantage of PVDF low acoustic impedance that allows an increase in the transmission of the acoustic power through impedance matching with the liquid medium, the measurement of the acoustic attenuation as a physical marker of bio cells was achieved.

Another interesting characteristic is the possibility of obtaining multifilament yarns made of PVDF, which offers the possibility to weave or knit it, making it suitable for the development of yarns that may be further used as threads for suturing purposes [189,190] with biocompatibility equivalent to other non-biodegradable polymeric material or as tubular conduits made of woven PVDF for vascular prostheses [191], or as a multifunctional tactile sensors for minimally invasive surgeries [192,193].

**Figure 2:**
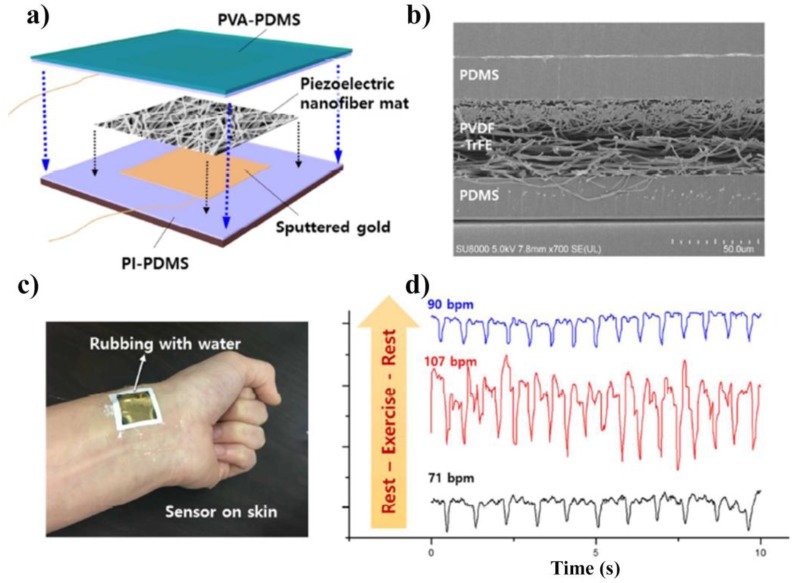
(**a**) Schematic illustration of a flexible piezoelectric sensor configuration including a P(VDF-TrFE) nanofibre mat sandwiched between a layer of PDMS and PVA-PDMS; (**b**) SEM image of the cross-section of the integrated sensor; (**c**) photographs of the integrated sensor applied on skin and (**d**) real-time sensor output waveforms at three consecutive body conditions before and after exercise. Reproduced with permission [196].

PVDF has also been used to produce ear prostheses due to its capability to respond to pressure and temperature, by using a stable multivibrator circuit, as changes in PVDF electrical response are observed according to variations of pressure and temperature [194]. Indeed, due to their pyroelectric and piezoelectric properties, PVDF-based prostheses, or prostheses with PVDF-based sensors, have been regarded as a promising tool for measuring temperature and pressure variations that could be translated to other applications such as health monitoring and human motion detection systems. Furthermore, PVDF electrospun yarns of nanofibres coated with poly(3,4-ethylenedioxythiophene) (PEDOT) allow the development of a highly sensitive, self-powered and wearable electronic skin [195]. Similarly, P(VDF-TrFE) was also used for the development of a skin-attachable mechanical sensor with high flexibility to be used as a wearable device, for medical monitoring systems (Figure 2) [196].

### 3.2. Tissue Engineering

In the last years, the piezoelectric properties of PVDF and its copolymers have been implemented in tissue engineering applications, once they can be used as smart materials, providing mechanical and/or electrical stimuli to cells and organs. This kind of approach becomes even more interesting since it is possible to find these stimuli in the human body. The piezoelectric properties can be found, for example, in bone, and many of the major functions in cells and organs of the human body are controlled by electrical signals [113].

Taking this into account, piezoelectric polymers have been used in several tissue engineering applications such as bone [113,197], muscle [13], neural [198] and cardiovascular [199] tissues, but also in wound healing [200] and blood vessel formation [201]. Particularly, depending on the pretended application, PVDF and copolymers have been used in different morphologies. However, most of the studies were performed in static conditions, demonstrating only the suitability of the materials. Therefore, only the studies that have been performed in dynamic conditions (with mechanical and/or electrical stimuli) will be presented, once the piezoelectric effect is only proven at these conditions [113].

**Figure 3:**
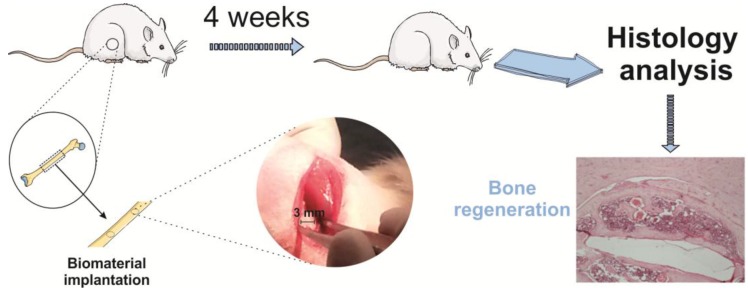
Graphical representation of the biomaterial implantation surgery protocol and representative section of Hematoxylin and Eosin (H&E) staining the healing defects 4 weeks after implantation on a poled β-PVDF film. Reproduced with permission [203].

Related to bone tissue engineering applications, it is possible to find several studies describing the use of PVDF as support for cell proliferation and differentiation under dynamic stimulation. Different kinds of cells were cultured on different PVDF films (poled and non-poled) showing different responses under dynamic conditions, namely under mechanical stimulation. Positively charged PVDF films showed high osteoblast adhesion and proliferation [202]. Negatively charged PVDF films [112] enhanced the osteogenic differentiation of human adipose stem cells. When mechanical stimuli are combined with biochemical stimuli, it leads to a successful approach of the biomimetic microenvironment present in the human body. These results were confirmed with in vivo studies, where PVDF films have been used to test their osteogenic properties in Wistar rats by analysing new bone formation in vivo in a bone defect [203]. After four weeks, significantly more defect closure and bone remodelling were observed (Figure 3). In this case, mechanical solicitations are obtained by rat movements.

In neural tissue regeneration, it is possible to find local electric fields during neural tissue development or regeneration [204]. These fields are created by electrically charged extracellular matrix materials that influence charge transfer reactions [204,205]. The use of piezoelectric polymers in this area offers the possibility to generate electrical stimuli without the need of additional energy sources. PVDF has been investigated for neural growth and differentiation in vitro and also in vivo [206]. The influence of the mechano-electrical stimulation in neural proliferation and the formation of neuron-like morphologies have been studied with PVDF films (with and without piezoelectric properties) [207]. For that, mechanical vibration (50 Hz) was applied to induce oscillating electrical fields at the film surface. After mechano-electrical stimuli, it was observed an increase of 115% of neuronal densities and 79% more neurites compared to the neurons grown in non-stimulated films. The findings were related to the electric stimuli generated by the mechanical vibration of the PVDF substrate. Higher vibration frequency (1200 Hz) has also been investigated and it was found that the local electrical charges enhance the nerve fibre outgrowth under mechanical strain on piezoelectric PVDF films [208]. The generation of electrical charges was also generated by acoustic stimulation in order to induce the neuritogenesis of PC12 cells [209]. The results indicate the activation of calcium channels through the dynamic PVDF stimulation by ultrasonic waves that induce the neurites generation via a cyclic adenosine monophosphate-dependent pathway. Additionally, the copolymer P(VDF-TrFE) has also been used. P(VDF-TrFE) piezoelectric fibrous scaffolds were used to study their influence in neural repair. It was observed that these scaffolds promote the formation of mature neural cells exhibiting neuron-like characteristics [204], while aligned fibres showed to promote primary neuron extension and directed the neurite outgrowth [198]. Tubular nerve guidance channels are built from P(VDF-TrFE) for neural regeneration. In this study, poled (negatively charged and positively charged) and unpoled channels were used. After four weeks, it was observed that the positively poled channels lead to greater number of myelinated axons in the regenerated nerves [210].

In muscle regeneration, the charge at the surface of PVDF films has already proven to influence cell proliferation [107]. However, until now, dynamic conditions, more precisely, mechanical and/or electrical stimuli, with piezoelectric biomaterials, have not yet been performed.

### 3.3. Microfluidic Systems

Microfluidic systems, also called lab-on-a-chip (LOC) or micro total analysis systems (μTAS), have gained increased attention in a broad range of applications because of their improved analytical performance through fast and accurate analysis using a reduced amount of sample and with less manual intervention [211]. The ability of handle fluids through microchannels is a key element of microfluidic systems, especially in the biomedical field where the pumping, mixing, reaction or even separation or sensing of fluids and/or compounds are required [212,213,214]. In many systems, piezoelectric ceramics with high piezoelectric response, such as PZT, are coupled to microfluidic systems with the main purpose of manipulating fluids or even particles [215,216]. Nevertheless, their lack of transparency does not allow their use in microfluidic systems that required optical detection and they are also rigid, dense and brittle and thus difficult to integrate. Alternatively, electroactive polymers, such as PVDF and P(VDF-TrFE), are potential substitutes due to their easy processability, easy integration, high mechanical strength, high flexibility, low acoustic impendence, high transparency in the visible spectral range, biocompatibility and low cost [215,217,218].

**Figure 4:**
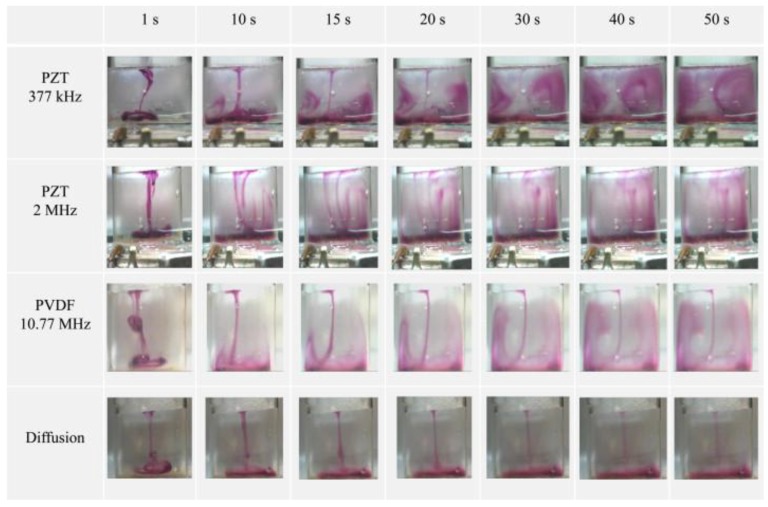
Movie frames of the experimental qualitative evaluation of a mixture between water and a dye with and without acoustic actuation. As proof of concept, a cuvette with dimensions of 8 mm width × 7 mm height × 2 mm thickness was used and actuated by PZT and β-PVDF transducers placed underneath, driven by a 24 Vpp sinusoidal voltage. Reproduced with permission [220].

Controllable and integrated piezoelectric pumps based on commercial piezoelectric PVDF films were integrated within a microfluidic chip made of polymethylmethacrylate (PMMA) [219]. The maximum flow rates of pumps were found to be linearly dependent of the viscosity of the fluid and values in the range of 0 to 300 μL·min^−1^ were obtained for water by controlling properly the alternating electrical signal applied on the diaphragm. The system was tested for the generation of droplet, being able to produce stable droplets with various sizes depending on the applied amplitude of the electrical voltage. A highly transparent piezoelectric transducer based on the layer-by-layer deposition of a piezoelectric P(VDF-TrFE) film and aluminium doped zinc oxide (AZO) electrodes on a glass substrate was designed and tested to generate acoustic waves, and consequently, the acoustic steaming phenomenon, that will promote the mixture of fluids inside microfluidic systems [139]. The efficiency of the piezoelectric actuator, located underneath the microfluidic system, was tested for the quantification of specific biomolecules in biological samples using optical detection. The reaction time required to quantify uric acid and nitrite, which involves the reaction between the biological sample that contains the biomolecules and specific reagents, was reduced in 23% and 32%, respectively, when compared to the reaction time achieved only by diffusion. These results demonstrate the potential of PVDF-based piezoelectric actuators to increase the efficiency in fluid mixing and thus to improve the overall performance of microfluidic systems that involve the mixture of fluids. Another study that compare the performance of two different piezoelectric materials, PZT and PVDF, to reduce the mixing time required in microfluidic devices was performed by numerical simulation and experimentally [220]. Both studies generated flow vortices and a considerable mixing time reduction above 90% for PZT and above 80% for PVDF (Figure 4).

In both studies referred previously, it was also observed an increase on the temperature of the transducer surface, and therefore inside the microfluidic system, which is advantageous for applications that require specific heating. A microfluidic pump that has the ability of being activated and controlled by a masked light source was also developed and it is based on an optopiezoelectric composite [221]. This composite is composed by a piezoelectric PVDF transducer with one of the electrodes replaced by a layer of titanyl phthalocyanine (TiOPc) photoconductive coating and an indium-tin-oxide (ITO) transparent electrode. The optopiezoelectric composite was implemented to verify the feasibility of creating two microfluidic pumps, where each pump can be optically turned on and off independently or simultaneously using a single driving source, and therefore reducing the size and number of driving units integrated into a microfluidic system. The performance of another microfluidic system integrating a P(VDF-TFE) diaphragm actuator was studied [218]. The system is composed by multilayers of active polymers films of about 1–2 μm thick, which allows to reduce the applied voltage from 1.8 kV to 100 V comparatively to using a single 20 μm thick P(VDF-TrFE) layer. Flow rate of 25 μL·min^−1^ was achieved using methanol as fluid, at 63 Hz with a backpressure of 350 Pa. The fabrication of an all inkjet-printed actuator based on P(VDF-TrFE) and electrodes printed from silver nanoparticles dispersions was also reported on the literature [222]. Printing technologies appear in the last years as promising manufacturing techniques due to their potential of highly flexible and additive material deposition on various substrates. The system demonstrates great potential to be used in microfluidic systems for presenting piezoelectric d_31_ coefficient in the range of 7 to 9 pm·V^−1^, allowing the generation of significant actuator deflection and with estimated pump rates of several hundred μL·min^−1^. A similar system based on successive inkjet printing of a P(VDF-TrFE) film sandwiched between two silver electrodes on a polyethylene terephthalate (PET) substrate was developed and characterized, demonstrating a piezoelectric d_31_ coefficient of 10 pm·V^−1^ and generated pumping rates up to 130 μL·min^−1^ [223].

Besides working as actuators, piezoelectric PVDF-based films have also been used as sensors in microfluidic systems. A flexible piezoelectric sensor based on patterned PVDF sheets was integrated in a PDMS microfluidic system to sense the flow rates, using the frequency amplitude response that varies with different flow rates [224]. The piezoelectric sensor was suitable to detect flow rates ranging from 100 to 450 L·min^−1^ at a high pressure on flexible PDMS substrates. A microfluidic end effector system consisting of a DC-microdiaphragm pump, one region of flexible latex tube, a PVDF sensor for in situ measurement of micro drag force and flow rates and a micropipette was also reported for fine handling and deposition of micro and nanoentities [225]. The setup shows a success rate for depositing carbon nanotubes between two electrodes close to 80%. Another interesting flow sensor is based on aligned P(VDF-TrFE) nanofiber films with 10 μm thick integrated in a microfluidic systems that was successfully used for real-time detection of low flow rates and viscosities [226]. This system composed by aligned P(VDF-TrFE) nanofibre films has the advantages of presenting larger specific surface area and higher sensitivity at lower pressure than P(VDF-TrFE) films. The flow sensor demonstrated ability to measure low flow rates ranging from 13 to 301 μL·h^−1^ with a sensitivity of 0.36 mV per 1 μL·h^−1^, and the highest electrical voltage difference of 120 mV at a flow rate of 451 μL·h^−1^. Moreover, the system was able to detect the viscosity of ethylene glycol aqueous solution in a dynamic flow with a sable output in the range from 1 to 16 mPa.s at 25 °C. 

### 3.4. Drug Delivery

Smart materials have raised strong interest for drug delivery applications. Through formulation strategies, encapsulation technologies and targeted approaches, drug delivery systems allow an increase in the fraction of drug that reaches its target (drug delivery to specific sites, organs, tissues or cells) enhancing the patient compliance, decreasing incidence of side effects, and improving selectivity of pharmacological activity [227]. Functional polymers offer an effective platform to achieve specific drug targeting, due to its ability to act as an intelligent drug delivery system that can sense and respond directly to pathophysiological conditions delivering the drugs at a controllable rate in a stable and biologically active form [228]. 

**Figure 5:**
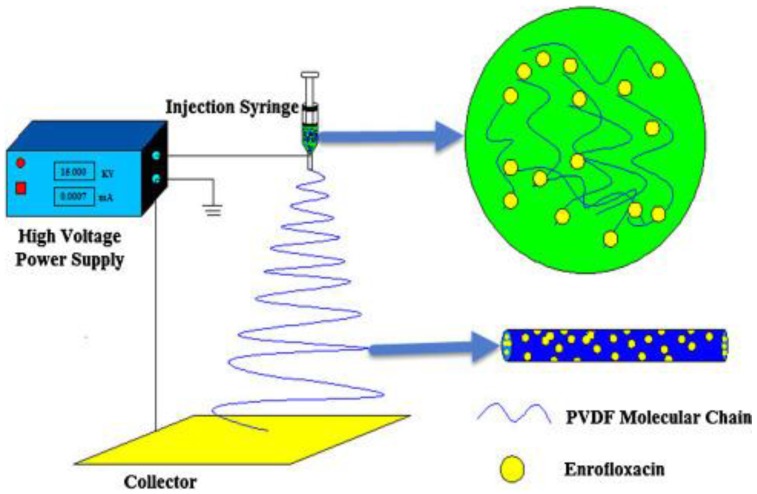
Preparation of drug-loaded PVDF fibres by electrospinning. Reproduced with permission [230].

To the best of our knowledge, few studies report on fluorinated polymers as drug carriers in drug delivery applications. Polymer membranes have been used as passive materials for drug release systems due to their capability to control the surface wettability from a hydrophilic to a hydrophobic state, and due to their reversible characteristic to turn to the initial state after the trigger removal [229]. PVDF porous membranes were developed for guided tissue regeneration, by grafting β-cyclodextrin (β-CD) onto the PVDF surface to form inclusion complexes with a large variety of drugs such as doxycyclin (DOX) and chlorhexidine (CHX) [134]. PVDF fibres loaded with enrofloxacin were also produced by electrospinning (Figure 5) for wound dressing applications [230]. Intelligent platforms based on neat and composite PVDF microspheres with magnetostrictive particles can be also used as an interesting approach to test in drug delivery systems [146,231]. Studies reveals that the application of a magnetic field allows to control the drug release kinetics in polymer/magnetostrictive particle composites [229].

## 4. Conclusions

Since their discovery during the 1930s, fluorinated polymers have been a subject of intense research for presenting a combination of useful and required properties in many technological applications, which mainly arise from their exceptional chemical resistance, high thermal stability, weather stability and high durability. In particular, fluorinated polymers showing smart properties, such as PVDF and its copolymers, represent a class of materials increasingly being applied, due to their ferroelectric, pyroelectric and, in particular, piezoelectric properties, i.e., its ability to convert electrical stimuli into mechanical response, and vice versa. This latter property, together with the excellent combination of chemical, physical and mechanical properties with the simple processability of PVDF-based polymers, has been increasingly investigated and applied in the biomedical field to develop highly instrumented devices, artificial muscle actuators, advanced tissue engineering and drugs delivery approaches, among others. Despite those advances which demonstrate the strong potential of this class of smart polymers, there is still a long road ahead of intense and dynamic research to obtain specific tailored properties that will definitely fulfil some of the most challenging (bio)technological applications in the near future. 

## Figures and Tables

**Table 1:** Typical properties of commercial fluorinated polymers.

	Perfluorinated			Partially fluorinated			
	PTFE	FEP	PFA	PCTFE	ETFE	PVF	PVDF
Monomer unit	TFE	TFE + HFP	TFE + PPVE	CTFE	Ethylene + TFE	VF	VDF
Density (g·cm^−3^)	2.16	2.15	2.15	2.10	1.70	1.40	1.75
Melting point (°C)	327	260	310	210	270	190	170
Continuous-use temperature (°C)	260	200	260	150	150	107	150
Tensile strength (MPa)	20–35	20–30	25–35	34–41	38–48	50–110	30–70
Refractive index	1.35	1.34	1.34	1.44	1.40	1.46	1.42
Dielectric constant at 1 MHz	2.1	2.1	2.1	2.6	2.5	8.5–11.0	8.4
Examples of typical applications	Membranes, wire and cable insulation, chemical resistant components	Chemical resistant components, cable insulation	Chemical resistant components, cable insulation	Packaging, barrier, sealing films	Moulded component, wire and cable insulation	Packaging, protecting films, solar panels	Sensors, actuators, wire and cable insulation
References	[16,17,18,19]	[20,21,22,23]	[23,24,25,26]	[27,28,29]	[30,31,32,33]	[34,35,36,37]	[13,38,39,40]

**Table 2:** Chemical structure of commonly used copolymers, some relevant electromechanical and dielectric properties [72] and their general applications.

Polymer	Chemical structure	Properties [72]	Applications
PVDF	–[CH_2_–CF_2_]_n_–	*d*_31_ (pC·N^−1^) = 8 to 22 *d*_33_ (pC·N^−1^) = −24 to −34 *k*_33_ = 0.20 ɛ′ = 6–12	Tissue engineering [108,112,113], membrane distillation [114], microcapsules for catalysis [115], membranes for rechargeable lithium metal cells [116], films for human-related sensor [117], wireless smart sensor for structural health monitoring [118,119]
P(VDF-TrFe)	–[CH_2_–CF_2_]_m_–[CHF–CF_2_]_n_–	*d*_31_ (pC·N^−1^) = 12 *d*_33_ (pC·N^−1^) = −38 k_33_ = 0.29 ɛ′ = 18	Tissue engineering [120], membrane separators for lithium ion batteries [121,122], transparent acoustic actuator and nanogenerator [123], thin film for pressure sensor for catheter application [124]
P(VDF-CTFE)	–[CH_2_–CF_2_]_m_–[CF_2_–CFCl]_n_–	*d*_31_ (pC·N^−1^) = 5.5% ^1^ *d*_33_ (pC·N^−1^) = −140 *k*_33_ = 0.39 ɛ′ = 13	Hydrophobic flat-sheet membranes for distillation and desalinization [125,126], membranes as lithium-ion battery separator [127]
P(VDF-HFP)	–[CH_2_–CF_2_]_m_–[CF_2_–CF(CF_3_)]_n_–	*d*_31_ (pC·N^−1^) = 30 *d*_33_ (pC·N^−1^) = −24 *k*_33_ = 0.36 ɛ′ = 11	Microporous membranes for membrane contactors for pervaporation [128], membrane distillation [129], battery separator [130,131,132]

*d*—Piezoelectric coefficient; *k*—Electromechanical coupling factor, ɛ′—Dielectric constant; ^1^ Longitudinal electrostrictive strain.

**Table 3:** Processing of PVDF and its copolymers for obtaining different morphologies.

Morphology	Method	Ref.
Films	Doctor blade	[147,148,149]
	Spin coating	[75,76,77,150,151,152]
	Printing	[153,154,155,156]
Porous films	Non-solvent induced phase separation (NIPS)	[78,135,157,158]
	Temperature induced phase separation (TIPS)	[149,159,160]
	Salt leaching	[144]
	Nylon template	
	Freeze extraction	
	Replica moulding	[145]
Fibres	Electrospinning	[136,161,162,163,164]
Microspheres	Electrospray	[146,165]
	Gelation	[166]
